# Progressive Supranuclear Palsy Diagnosed After a Severe Fall Trauma in a Patient Who Experienced Episodes of Easy Falling

**DOI:** 10.7759/cureus.59643

**Published:** 2024-05-04

**Authors:** Yuji Kaneko, Eisuke Furui, Hiroki Isono

**Affiliations:** 1 Medicine, Hokkaido University School, Hokkaido, JPN; 2 Neurology, HITO Medical Center, Ehime, JPN; 3 General Medicine, HITO Medical Center, Ehime, JPN

**Keywords:** parkinsonism, magnetic resonance imaging (mri), trauma, easy falling, progressive supranuclear palsy

## Abstract

Progressive supranuclear palsy (PSP) is characterized by parkinsonism, downward gaze disorder, and a tendency to fall due to degeneration of the basal ganglia, the brain stem, and the cerebellum. We report a case of PSP that was diagnosed following a traumatic hemopneumothorax caused by a fall while descending stairs. A 79-year-old man experienced lightheadedness and frequent falls for two years. He fell on stairs at home and was transferred to our hospital due to mobility issues. He was hospitalized and treated for traumatic hemopneumothorax. Neurological examination revealed vertical ocular motility disorder, positive Myerson's sign, increased muscle stiffness, and increased limb tendon reflexes. Brain MRI showed a hummingbird sign. In this case, a midbrain area of 58.1 mm^2^ was consistent with PSP. He had no medication history that could have caused falls. He was diagnosed with PSP based on clinical and imaging findings, and treatment with levodopa was initiated. Two months later, walking showed limited improvement, and living at home became difficult. He was discharged to a care facility. PSP is a risk factor for frequent falls in the elderly. PSP usually requires three to four years for diagnosis, although falls appear earlier than in other forms of degenerative parkinsonism. Additionally, PSP often results in repeated dynamic falls due to a decreased perception of danger associated with reduced frontal lobe function. As a result, the severity of trauma from falls in PSP tends to be higher than in other neurodegenerative diseases. Therefore, early diagnosis of PSP may help improve patients' quality of life and prevent trauma. Despite frequent falls over two years, the cause was not thoroughly investigated until the patient experienced severe trauma. The lesson from this case is the importance of a thorough neurological examination and sagittal MRI for elderly patients experiencing repeated falls, to consider the possibility of PSP. Furthermore, quantitative evaluation of MRI enhances the diagnostic accuracy of PSP.

## Introduction

Progressive supranuclear palsy (PSP) is characterized by parkinsonism, downward gaze disorder, and a tendency to fall due to degeneration of the basal ganglia, the brain stem, and the cerebellum [1]. In the early stages, distinguishing parkinsonism from other related disorders, such as corticobasal degeneration and multiple system atrophy, is challenging due to a lack of specific clinical findings [2,3]. PSP is a cause of frequent falls in the elderly. Additionally, falls related to PSP are more likely to result in severe trauma [3,4]. Parkinson's drugs like levodopa (L-DOPA) and dopamine agonists are employed in treating PSP, however, their effectiveness is limited, and the progression is faster than other neurodegenerative disorders causing parkinsonism [5]. Therefore, early diagnosis of PSP may contribute to improving patients' quality of life and preventing trauma. We report a case of PSP that was diagnosed following a traumatic hemopneumothorax caused by a fall during a stair descent.

## Case presentation

**Figure 1:**
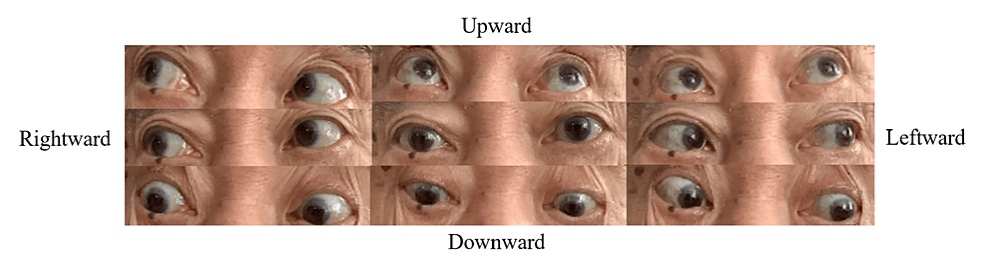
Patient's eye movements in 9 directions He had an upward ocular motility disorder.

For two years, a 79-year-old man endured bouts of lightheadedness and frequent falls without much concern. He suffered a fall on the stairs at his residence, resulting in severe trauma that necessitated his transfer to our hospital. Notably, he did not lose consciousness during the fall. He was hospitalized and underwent thoracic drainage for traumatic hemopneumothorax. On admission, the patient was conscious and clear and had no significant neurological abnormalities. Subsequently, we actively conducted a close examination of the factors contributing to his frequent falls. His medical history included neurogenic bladder, benign prostatic hyperplasia, chronic kidney disease, hypertension, dyslipidemia, and chronic gastritis. Oral medications were silodosin, dutasteride, and bethanechol. His life history revealed independence in activities of daily living (ADL) and unassisted walking with a cane. Vital signs were temperature 37.0°C, blood pressure 124/69 mmHg, pulse rate 58 beats/min, and SpO2 96% (room air). Physical examination revealed clear consciousness and a good score of 26 on the revised Hasegawa Brief Cognitive Rating Scale, which is used as a screening test for dementia. Visual acuity was normal, as was the visual field. Pupil diameter and light reflex were normal. He had an upward ocular motility disorder (Figure 1).

He had a mask-like facial expression with decreased blink frequency and abnormal reflex blinking during the hand-blindness test. Hearing was normal. A manual muscle test (MMT) revealed no significant muscle weakness. There was a predominant rigidity of the distal muscles of the extremities. He had no neck stiffness. The patient had a normal sense of touch, warmth, and pain. He also had a positive Myerson's sign, hyperreflexia of the tendon reflexes of the extremities, and positive Babinski's sign bilaterally. The finger-nose-finger test was normal. He exhibited a flexed posture with a rounded back and no tremors. The pull test to check for postural retention disorder scored unified Parkinson’s disease rating scale (UPDRS) 3, and postural retention was abnormal. His gait showed a small step length, while the step distance remained normal (Video 1).

**Video 1:** Walking shot from the side He exhibited a flexed posture with a rounded back. His stride was constant with a small gait.

**Figure 2:**
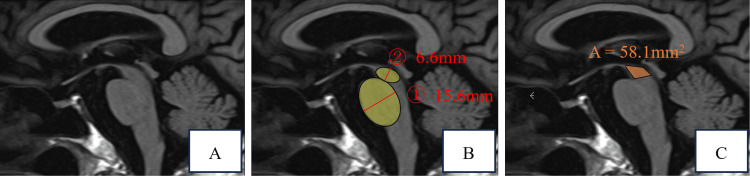
Sagittal T1 image on MRI (A) A hummingbird sign was seen because of the atrophy of the mesencephalic tegmentum. This sign is a characteristic MRI finding in PSP. It is observed in sagittal head MRI when the midbrain tegmentum atrophies. (B) ① Anterior-posterior midbrain diameter　② Anterior-posterior pons diameter Elliptical regions of interest were placed over the pons and the midbrain in the midsagittal slice. The maximal measurement perpendicular to the major axis was taken. The posterior border of the pons was clearly identifiable and did not include the pontine tegmentum. The midbrain measurement did not include the collicular plate. (C) Midbrain area A  straight line is drawn from the lowest part of the interpeduncular fossa to the lowest part of the corpora quadrigemina. This line is the boundary between the midbrain and the pons.

He did not exhibit any autonomic nervous system disorders, such as dysuria or constipation. Blood tests revealed no significant findings. Sagittal head magnetic resonance imaging (MRI) revealed the characteristic hummingbird sign in the PSP, indicative of midbrain tegmentum atrophy (Figure 2A). Magnetic resonance angiography (MRA) of the head showed no significant findings. The midbrain's anteroposterior diameter was 6.6 mm, with a midbrain-to-bridge ratio of 0.42. Measurements were taken as shown in Figures 2B and 2C.

The midbrain area of this case was 58.1 mm2. We excluded ADL decline, environmental factors, or medications as contributors to the patient's frequent falls. We first diagnosed parkinsonism based on the positive Myerson's sign and gait status. We ruled out vascular, toxic, or drug-induced parkinsonism based on MRA results, the patient's history, and medications. We considered Parkinson's disease, corticobasal degeneration, spinocerebellar degeneration, Lewy body dementia, and PSP in the differential diagnoses. He was diagnosed with PSP based on clinical findings such as vertical ocular motility disorder and increased muscle stiffness, as well as head MRI findings. Treatment with levodopa/carbidopa and rehabilitation was started. At the two-month point of treatment initiation, there was limited improvement in walking, and living at home became difficult. Consequently, the decision was made for him to be admitted to a care facility.

## Discussion

This was a case of PSP diagnosed following a traumatic hemopneumothorax associated with a fall. Two important lessons were learned from this case.

First, in elderly individuals with frequent falls, we should consider PSP as a potential diagnosis. PSP increases the risk of early falls compared to other forms of degenerative parkinsonism [6]. However, diagnosing PSP typically takes longer, approximately three to four years from the initial onset of clinical symptoms [7,8]. This is attributed to a complex array of neurological findings. In addition, PSP often results in repeated dynamic falls due to decreased perception of danger associated with reduced frontal lobe function. As a result, the severity of trauma from falls tends to be higher in PSP than in other neurodegenerative diseases [3,4]. Therefore, early diagnosis contributes to improving patients' quality of life and preventing trauma [3]. In this case, despite frequent falls over two years, the cause was not thoroughly investigated until the patient experienced severe trauma. The reason for the delay in diagnosis is that the patient was unaware of the disease and did not seek medical care for the primary complaint of easy falls.

Secondly, utilizing the measurement function of MRI has enhanced the diagnostic accuracy of PSP. One imaging characteristic of PSP is the "hummingbird sign," resulting from midbrain tegmentum atrophy in MRI sagittal sections [9]. However, this imaging feature relies on visual judgment, and there are no defined criteria for its determination. Therefore, non-specialists have particular difficulty in making a judgment. Massey et al. reported that the anteroposterior diameter of the midbrain or the midbrain/bridge anteroposterior diameter ratio in the midsagittal section of T1-weighted MRI images allows for a quantitative diagnosis of PSP [10]. The anteroposterior diameter is the measurement of the anterior-posterior axis of the midbrain (excluding the midbrain lid) and the base of the bridge, each represented by a cross-shaped ellipse along their long axes. Setting the cutoff value for the midbrain's anteroposterior diameter at 9.35 mm results in a test sensitivity of 83% and specificity of 100%. Also, setting the cutoff value for the midbrain/bridge ratio at 0.52 yields a test sensitivity of 67% and specificity of 100%. Additionally, Oba et al. reported that measuring the midbrain area is effective, with PSP typically less than 70 mm2 [11]. In this case, an anteroposterior midbrain diameter of 6.6 mm, a midbrain/bridge diameter ratio of 0.42, and a midbrain area of 58.1 mm2 were consistent with PSP. Quantitative evaluation of MRI images can enhance the accuracy of PSP diagnosis in cases where visual determination of midbrain atrophy is challenging.

## Conclusions

PSP is a high risk for severe trauma. The lesson to be learned from this case is the importance of a thorough neurological examination and sagittal MRI for elderly patients experiencing repeated falls, to consider the possibility of PSP. Furthermore, quantitative evaluation of MRI imaging enhances the diagnostic accuracy of PSP.
